# ﻿Two new cichlid species of the genus *Labrochromis* from rocky reefs of Lake Victoria, Tanzania (Perciformes, Cichlidae)

**DOI:** 10.3897/zookeys.1240.125699

**Published:** 2025-06-05

**Authors:** Anna Mahulu, Ole Seehausen

**Affiliations:** 1 Division of Aquatic Ecology and Evolution, Institute of Ecology and Evolution, University of Bern, Bern, Switzerland University of Bern Bern Switzerland; 2 Department of Fish Ecology and Evolution, Centre of Ecology, Evolution and Biogeochemistry, EAWAG Swiss Federal Institute of Aquatic Science and Technology, Kastanienbaum, Switzerland Centre of Ecology, Evolution and Biogeochemistry, EAWAG Swiss Federal Institute of Aquatic Science and Technology Kastanienbaum Switzerland

**Keywords:** Haplochromine, Mwanza Gulf, pharyngeal crusher, Speke Gulf, taxonomy

## Abstract

Lake Victoria is home to a unique and taxonomically understudied species flock of endemic haplochromine cichlid fishes, with many morphologically specialized trophic groups and many different species in each of them. One of several mollusk-eating trophic groups are the pharyngeal snail-crushers of the genus *Labrochromis* Regan, 1920. Currently, six species from Lake Victoria have been described in this genus, none of which occupies rocky shores and reefs. Rocky shores and reefs of Lake Victoria, however harbor rich assemblages of habitat-specialized cichlids and these include snail-crushers. Here two new species of *Labrochromis* are described from this habitat in the Southeastern part of Lake Victoria. These species are distinct in their ecology, morphology, and male nuptial coloration from all previously described *Labrochromis* species, and they are distinct from each other in oral dentition, morphology, and stripe pattern. These species are named *Labrochromismawe***sp. nov.** and *Labrochromismawepili***sp. nov.** Both are currently only known from the Mwanza and Speke Gulf regions of the lake in Tanzania.

## ﻿Introduction

The world’s largest tropical lake by surface and the largest in the African great lake’s region, Lake Victoria, hosts a remarkable species-flock comprising ~ 500 cichlid species (Genner et al. 2004; Witte et al. 2013). This diverse array exhibits a spectrum of morphologies, melanic stripe patterns, male nuptial coloration, diets, behaviors, and habitat preferences (Greenwood 1974; Seehausen 1996; Santos et al. 2023). Remarkably, this diversification primarily occurred within the last ~ 15,000 years (Meier et al. 2017, 2023), establishing the haplochromine cichlids of Lake Victoria as a paradigmatic example of explosive evolution and adaptive radiation among vertebrate animals (Seehausen 2006; Meier et al. 2017; McGee et al. 2020).

Despite its very recent origin (Meier et al. 2017, 2023), the ecological diversity of Lake Victoria’s cichlid species flock, in terms of trophic groups and habitat guilds, is on par with that of the older species flocks in lakes Malawi and Tanganyika (Greenwood 1974; Seehausen 1996). Species richness in this young lake is higher than in the older Lake Tanganyika, which though exhibits larger morphological diversity (Young et al. 2009; Martinez et al. 2024).

Contrary to long-held beliefs that rocky environments were not an important habitat for cichlids in Lake Victoria (Fryer and Iles 1972; Greenwood 1984; Goldschmidt and Witte 1992), more recent studies have unveiled a species-rich rock-dwelling cichlid assemblage, comparable in ecology and diversity to that in Lake Malawi (Seehausen 1996; Seehausen et al. 1998). In spite of some initial efforts resulting in the description of 15 of the newly discovered rock-dwelling species (Seehausen et al. 1998), the vast majority remained undescribed, emphasizing the need for continued taxonomic work on this rich assemblage. Here we make a start by describing new snail-eating members.

Lake Victoria harbors a much greater diversity of snail-eating cichlids than any of the other much older African Great Lakes (Seehausen 1999). These snail-eating cichlids can be broadly categorized into two distinct groups with different feeding strategies and adaptations. The first group, known as oral shellers and oral crushers, possess specialized oral dentition that enables them to either extract the soft body of the snail from its shell or to crush the shell using their oral jaws (Witte and van Oijen 1990). The second group, referred to as pharyngeal crushers, is characterized by massively enlarged pharyngeal jaws equipped with broad molariform teeth that allow these fish to crush the hard shells of *Melanoides* snails. Greenwood considered this group a distinct phylogenetic lineage, the genus *Labrochromis*. This genus comprises currently six described species from Lake Victoria. These are *Labrochromisishmaeli* (Boulenger, 1906), *L.humilior* (Boulenger, 1911), *L.pharyngomylus* (Regan, 1929), *L.teegelaari* (Greenwood & Barel, 1978), *L.mylergates* (Greenwood & Barel, 1978), and *L.ptistes* (Greenwood & Barel, 1978). ‘*Haplochromistheliodon*’ shares some derived features with *Labrochromis*. Specifically, the extent to which the lower pharyngeal bone and its dentition are hypertrophied is comparable to that of *L.humilior*. However, the neurocranial apophysis for the upper pharyngeal bones in ‘*H.’ theliodon* is not as well developed as in *L.humilior* and the chest scales of ‘*H.’ theliodon* are significantly smaller compared to those of *L.humilior*. Based on these characteristics, Greenwood (1980) refrained from placing ‘*H.’ theliodon* in the genus *Labrochromis*. Given that the new species we describe here are rocky shore dwellers and have small deeply embedded chest scales, we include ‘*H.theliodon*’ in our comparison and differential diagnosis irrespective of that Greenwood did not assign it to *Labrochromis*. Because we had no opportunity to study Greenwood’s material, we do not assign ‘*H.theliodon*’ to *Labrochromis* but use ‘*Haplochromis*’ in quotation marks following Greenwood to reflect the uncertainty regarding the generic placement of this species.

Additionally, Greenwood had assigned one species of Lake Edward to *Labrochromis*, *L.mylodon*. Phylogenomic work recently revealed a clade containing all highly specialized pharyngeal snail crushers of the Lake Victoria radiation for which whole genome sequences were available, including described *Labrochromis* species, but the phylogenetic distinctiveness of all the haplochromines of Lake Edward that form a separate radiation remains evident (Meier et al. 2023). The Lake Edward snail crusher should therefore probably not be considered as a member of the genus *Labrochromis*.

Here we describe two new species of pharyngeal snail-crushers from Lake Victoria in the genus *Labrochromis*. Greenwood (1980) redescribed *Labrochromis* Regan, 1920 as a genus of haplochromines characterized by a massive hypertrophy of the pharyngeal mill. This hypertrophy is especially prominent in the lower pharyngeal bone and its dentition. The degree of hypertrophy is the sole derived morphological character that distinguishes *Labrochromis* from other lineages with generalized facies within the Lake Victoria radiation. As the fish grows, the apophysis becomes relatively more massive than in other species (Greenwood 1980). The oral jaw teeth of *Labrochromis* are moderately stout, slightly recurved, and have a subcylindrical neck with a crown that is not markedly compressed. The teeth in the outer row of the oral jaws are of the basic bicuspid type in small individuals, but weakly bicuspid and some unicuspid teeth occur in specimens of all species beginning at a length of ~ 70–80 mm standard length (SL). The proportion of unicuspid teeth in the outer tooth row increases with the length of the fish, and fishes larger than 100 mm SL have mostly unicuspid and weakly bicuspid teeth while exclusively unicuspid dentition is uncommon even in large fish. The inner row teeth are small, tricuspid and are arranged in 1–3 (rarely 4) rows anteriorly and anterolaterally in both jaws. Despite their typically generalized facies, most species of *Labrochromis* have a “heavy headed” and deep bodied appearance (Greenwood 1980). The fish have a horizontal or slightly oblique mouth with isognathous jaws, and their lips are not thickened. The premaxilla is not produced into a beak. The dentary, which carries the lower jaw teeth, is slender and shallow, and the lower jaw length ranges from 34–44% of the head length (HL) (modal range 37–40%).

While these morphological traits provide some clarity in genus assignment, as in any very species rich group, there are challenges in morphologically delimiting the genus from some forms assigned to other, phylogenomically not closely related genera due to overlapping values in individual traits and possibly parallel evolution. Hoogerhoud (1984) highlighted these difficulties and expressed doubts about the validity of the genus *Labrochromis*. He especially emphasized the lack of a morphological trait gap between *Labrochromis* and *Gaurochromis*, but Hoogerhoud analyzed traits as univariate characters, and these genera have been shown to form distinct clusters when Seehausen (1996) subjected Hoogerhoud’s data to a multivariate cluster analysis. These genera also emerged as distinct and not closely related in recent phylogenomic work (Meier et al. 2023). For these reasons and additional reasons given by Seehausen (1996), we follow the classification proposed by Greenwood (1980) which doubtlessly aids a more nuanced understanding of species diversity and evolutionary relationships within Lake Victoria’s cichlid fish than lumping 500 species into a single genus. A larger number of previously unknown rock-dwelling haplochromines were introduced to science in 1996 (Seehausen 1996). The two new species that we describe here, were part of this largely new fauna and were designated in 1996 with provisional names *Labrochromis* sp. “stone” (first introduced in Witte et al. 1992), and *Psammochromis* sp. “striped crusher”. That the latter was mistaken as a member of the genus *Psammochromis* is likely explained by the circumstance that only subadult individuals (<93 mm SL; compared to a maximum size of 144 mm SL reported here) were available in which the pharyngeal apparatus and dentition were incompletely developed (Seehausen 1996). The former is distributed in central/northern Mwanza Gulf where it is known from at least 13 sites. The latter is only known from rocky shores and offshore reefs within the Speke Gulf, notably Makobe, Ruti and Igombe Islands, and from Hippo Island in the northern Mwanza Gulf. The sympatric coexistence of these two species is rare and has only been observed at Hippo Island in the northern Mwanza Gulf (Seehausen 1996).

## ﻿Materials and methods

**Figure 1.**
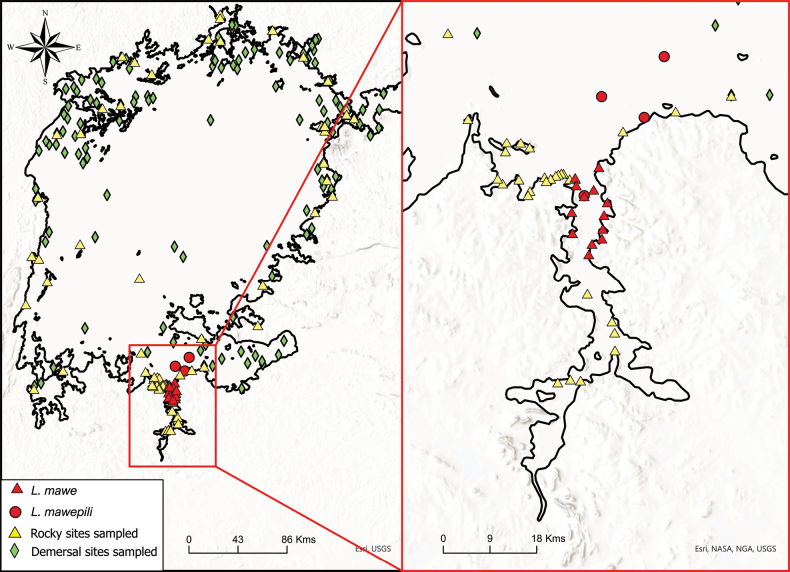
Known occurrences of *Labrochromismawe* sp. nov. and *Labrochromismawepili* sp. nov. against the background of all sites sampled in Lake Victoria by the authors’ team. Rocky sites are characterized by rocky substrates, which include boulders of various sizes, slabs and blocks, and occasionally large pebbles. Demersal sites are non-rocky substrates, mostly soft bottom, sometimes sand. Each rocky site was sampled with a comparable fleet of gillnets of various mesh sizes (1–2 inches stretched mesh) set in the morning, usually between 8 and 9 am and pulled after 4–5 hours. Each demersal site was trawled for ten minutes, which amounts to a stretch of ~ 1 km long and 10 m wide.

All specimens were collected in Mwanza and Speke gulfs, Lake Victoria, Tanzania between 1993 and 2010 with gill nets. We base the description of *Labrochromismawe* sp. nov. on specimens from Python Island and Kissenda Island, and that of *Labrochromismawepili* sp. nov. on specimens from Makobe Island. We refer to our specimens from these three islands as three populations that we use for the purposes of comparisons. The occurrence of *Labrochromis* species in rocky habitats has been assessed for more than 100 islands and rocky mainland shores all around the lake, but no positive records of either of these two species were made outside the above-described regions (Fig. 1). While confidence in the absence of our two species from any one site is limited given the modest sampling effort for many of the individual sites, the total absence in records across all of these sites outside the Mwanza Gulf and Speke Gulf regions suggests that these species may indeed be regionally endemic to relatively small parts of the lake. This is relatively unusual for Lake Victoria cichlids (Seehausen 1996; Genner et al. 2004). Neither of the two species has been recorded in trawl shots over soft bottom anywhere in the lake either (Fig. 1) and seem to be confined to rocky shores.

**Figure 2.**
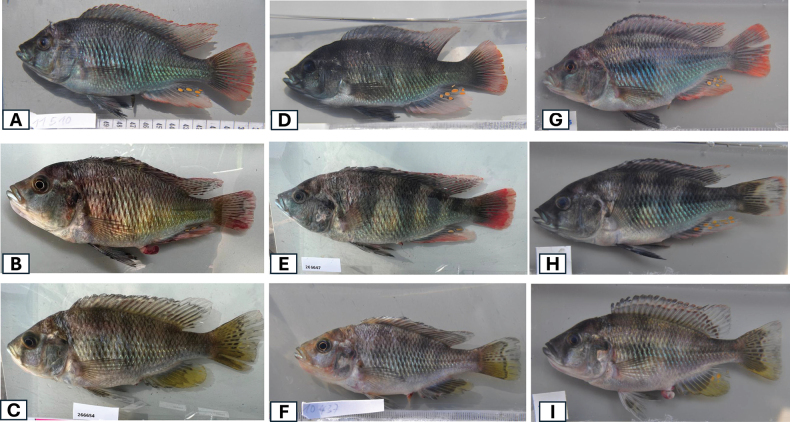
From top left **A** male *Labrochromismawe* sp. nov. blue morph Python Island **B** male *Labrochromismawe* sp. nov. red morph Python Island and **C** female *Labrochromismawe* sp. nov. Python Island **D, E** two males *Labrochromismawe* sp. nov. Kissenda island and **F** female *Labrochromismawe* sp. nov. Kissenda Island **G, H** two males *Labrochromismawepili* sp. nov. Makobe island and **I** female *Labrochromismawepili* sp. nov. Makobe island.

After being captured, some individuals were photographed alive in photo cuvettes to document their live coloration (Fig. 2). All specimens were anesthetized with an overdose of MS222 and subsequently kept on melting ice until their fixation in a neutralized solution consisting of 6% formaldehyde back in the laboratory. The specimens were later transported to the Swiss Federal Institute of Aquatic Science and Technology (**Eawag**). The final processing for long-term storage involved rinsing under flowing water to remove the formalin, followed by stepwise transfer to ethanol of increasing concentrations and final storage in 75% ethanol. All type material was deposited at the Natural History Museum of Bern (**NMBE 1111864–NMBE 1111917**).

The sex of the specimens was determined by observing the coloration of the freshly caught fish and examining their genital papillae. We performed a full head CT scan at 10 µm resolution for comparative morphology, including dentition. We scanned representative specimens of each population (see below) The CT scanning was performed using SkyScan2214 v. 1.8 at the microscopy imaging center of the University of Bern, Switzerland. We used 3D slicer software (Fedorov et al. 2012) to extract the oral and pharyngeal jaws from each head scan. We assessed the shape of teeth in the outer row of both oral jaws following categories of (Barel et al. 1977) and coded as 1 = equally bicuspid, 2 = sub equally bicuspid, 3 = unequally bicuspid, 4 = weakly bicuspid, 5 = unicuspid (Seehausen 1996). The number of inner tooth rows was counted at the anterior most part of both jaws. The number of dorsal fin spines, scales in the lateral line, dorsal fin rays, anal fin spines, anal fin rays, pelvic fin spines and pelvic fin rays were also counted following Barel et al. (1977).

Morphometric measurements were taken to the nearest 0.1 mm using digital calipers with needles glued to the tips. We measured 16 informative linear distances (Table 1) following (Barel et al. 1977): standard length (**SL**), body depth (**BD**), head length (HL), head width (HW), lower jaw length (**LJL**), lower jaw width (**LJW**), snout length (**SnL**), snout width (**SnW**), eye length (**EyL**), eye depth (**EyD**), cheek depth (**ChD**), pre-orbital depth (**POD**), inter-orbital width (**IOW**), pre-orbital width (**POW**), caudal peduncle length (**CPL**), and caudal peduncle depth (**CPD**). We measured each trait thrice and checked for outliers. If the deviation between any one of the three measurements and the other two exceeded 5%, we repeated the measurement and replaced the outlier with the new measurement. Finally, we calculated the mean of the three measurements. For some analyses we converted measurements into percentages relative to reference measurements such as SL or HL. For other analyses, we worked with the residuals of linear regressions of each trait against standard length.

**Table 1.** Morphometric, dentition and meristic characters of *L.mawe* sp. nov. and *L.mawepili* sp. nov. Samples from the three populations and sexes are indicated separately. Island is abbreviated as Isl.

Morphometric, dentition and meristic characters	Males, Python Isl. (n = 13) *L.mawe* sp. nov.		Females, Python Isl. (n = 4) *L.mawe* sp. nov.		Males, Kissenda Isl. (n = 10) *L.mawe* sp. nov.		Females, Kissenda Isl. (n = 7) *L.mawe* sp. nov.		Males Makobe Isl. (n = 12) *L.mawepili* sp. nov.		Females, Makobe Isl (n = 5) *L.mawepili* sp. nov.	
	Range	Mean±SD	Range	Mean±SD	Range	Mean±SD	Range	Mean±SD	Range	Mean±SD	Range	Mean±SD
SL (mm)	77.6–127.8	107.0±15.8	81.6–116.2	97.8±13.5	82.8–124.3	102.6±13.4	99.1–120.0	106.9±8.0	88.2–144.2	115.2±17.1	80.7–120.8	108.5±16.3
BD%SL	37.2–44.0	40.8±2.8	36.9–41.9	39.1±2.0	36.1–42.8	39.7±1.9	37.2–39.2	38.4±0.8	34.5–42.7	38.5±2.7	35.0–39.5	36.8±2.0
HL%SL	31.7–34.2	33.3±0.7	31.8–34.9	33.4±1.1	32.0–35.0	33.5±1.1	32.6–35.8	34.3±1.3	32.6–37.0	34.4±1.1	33.6–36.3	34.4±1.1
HW%SL	16.0–18.9	17.5±0.9	16.8–18.4	17.5±0.6	17.2–19.1	18.1±0.6	16.3–18.5	17.4±0.6	16.0–18.9	16.9±0.8	16.0–18.9	17.5±0.7
CPL%SL	14.1–17.7	16.0±1.1	14.3–17.6	15.8±1.2	14.9–17.2	15.7±0.8	14.6–16.4	15.6±0.5	15.2–17.5	16.4±0.7	15.8–18.0	16.6±0.9
CPD%SL	10.9–13.5	12.2±0.5	11.4–12.6	12.0±0.5	11.4–13.5	12.3±0.6	11.3–12.1	11.6±0.3	9.5–12.9	11.5±1.0	10.3–11.9	10.7±0.8
SnL%HL	27.8–36.7	30.9±2.6	27.9–35.8	30.4±2.9	27.5–33.5	30.2±1.7	27.3–32.3	29.7±1.9	30.7–36.0	32.5±2.2	30.2–32.4	31.4±0.7
SnW%HL	27.2–34.0	30.7±2.2	24.3–33.8	29.9±3.3	21.7–32.4	28.2±3.1	27.2–31.5	29.4±1.4	25.9–31.7	29.3±2.3	25.7–28.8	27.2±1.4
POD%HL	16.1–20.3	17.2±1.2	15.4–18.5	17.3±1.1	16.0–21.2	18.5±1.9	16.2–22.1	19.2±2.2	17.0–19.7	18.1±0.9	16.8–18.8	17.7±0.8
POW%HL	29.4–39.0	34.2.±2.9	29.3–36.2	32.2±2.4	30.4–32.2	32.2±1.4	28.4–35.7	33.4±3.6	27.6–32.5	30.3±1.2	28.5–31.4	30.0±1.1
EyL%HL	22.6–25.1	24.4±1.5	23.7–25.9	24.6±1.4	21.8–25.7	24.2±1.8	21.7–24.9	23.9±1.1	18.7–25.1	23.4±2.4	19.2–25.8	21.8±3.1
EyD%HL	20.9–27.7	23.5±2.0	21.4–26.7	24.4±2.1	19.8–25.9	24.2±2.7	20.1–24.9	22.8±1.6	19.7–24.6	22.9±1.5	20.5–25.5	22.8±2.0
ChD%HL	21.7–29.3	24.1±2.0	22.4–27.5	22.2±2.4	23.0–28.3	24.4±3.6	25.4–29.2	25.8±2.8	24.2–30.1	26.7±2.6	25.7–29.5	26.7±2.5
LJL%HL	35.2–43.2	39.5±2.3	36.7–44.2	40.1±2.9	35.7–43.8	40.5±2.7	33.1–40.2	38.1±2.6	29.9–41.4	38.2±2.9	38.2–42–0	40.40±1.5
LJW%HL	24.9–33.8	29.7±2.9	22.7–30.3	27.1±3.0	26.7–34.9	29.7±3.2	25.9–33.0	27.5±1.9	20.2–26.9	24.6±2.1	22.8–27.5	25.7±1.8
IOW%HL	25.0–30.2	28.2±1.6	26.0–29.4	27.3±1.3	26.8–31.8	28.1±1.6	24.0–30.4	27.3±2.1	23.3–27.9	25.40±1.5	23.6–27.3	24.6±2.0
EyD/EyL	0.9–1.0	0.9±0.4	0.9–1.0	0.9±0.0	0.84–1.0	0.9±0.4	0.9–1.0	0.9±0.1	0.9–1.0	0.9±0.1	0.9–1.0	0.9±0.1
LJL/LJW	1.1–1.8	1.6±0.2	1.3–1.7	1.5±0.1	1.22–1.7		1.2–1.7	1.4±0.1	1.3–1.8	1.6±0.2	1.4–1.8	1.7±0.2
Outer tooth cusp shape	4–5		4–5		4–5		4–5		4–5		4–5	
No. of inner tooth rows	2		2		2		2		2		2	
Scales in Lateral line	31(7) 32(4)		31(4)		31(6) 32(2)		31(4) 32(1)		32(4) 33(6)		33(4)	
Dorsal fin spines	15(11)		15(4)		15(8)		15(5)		15(10)		15(4)	
Dorsal fin rays	9(11)		9(4)		9(8)		9(5)		9(9) 10(1)		9(4)	
Anal fin spines	3(11)		3(4)		3(8)		3(5)		3(10)		3(4)	
Anal fin rays	9(11)		9(4)		7(8)		7(5)		7(1)8(9)		8(4)	
Pelvic fin spines	1(11)		1(4)		1(8)		1(5)		1(10)		1(4)	
Pelvic fin rays	5(11)		5(4)		5(8)		5(5)		5(10)		5(4)	

For univariate analysis of trait variation between species and populations we performed Kruskal-Wallis tests (kruskal.test function) on the proportional measurements followed by pairwise post-hoc comparisons using Dunn’s test (dunn.test function). To control for multiple comparisons, we applied a Bonferroni adjustment within each trait, dividing the alpha level (α = 0.05\alpha = 0.05α = 0.05) by the number of pairwise comparisons for that trait.

To visualize the total variation across groups, the dimensionality in the residual trait data was reduced using principal component analysis (PCA). We used the prcomp() function in the base stats package in R. We employed a multivariate analysis of variance (MANOVA) to assess the statistical significance of differences between groups in the principal component (PC) scores. Principal component analysis is frequently employed in taxonomic studies of haplochromine cichlids to evaluate if groups are different in multivariate space (Katongo et al. 2017; Vranken et al. 2020, 2023). To facilitate the identification of distinctive features for species identification, we subsequently performed Discriminant Function Analysis (DFA) on the three populations using the lda() function from the MASS package in R. We used R v. 4.1.2 (2021-11-01) for all analyses.

For species delineation broadly speaking, we looked for coincidence between differences among groups in morphological traits and differences in melanin pattern and/or male nuptial coloration (Seehausen et al. 1998). This combination of traits has proven to produce reliable species delineation, as has been found in all speciation genomics studies of cichlid fish from Lake Victoria to date (Keller et al. 2013; Wagner et al. 2013; Van Rijssel et al. 2022; Meier et al. 2023). To test for intraspecific sexual dimorphism in morphometric traits, we conducted Mann-Whitney U tests on each trait separately. The significance level was set at α = 0.05.

## ﻿Results

**Figure 3.**
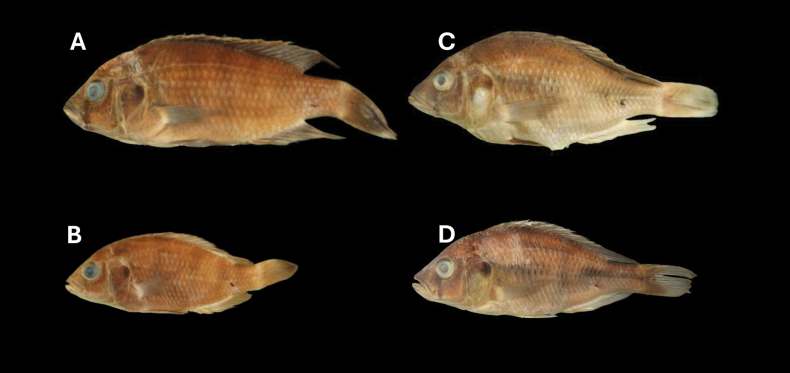
**A***Labrochromismawe* sp. nov. holotype, NMBE 1111880, male, 127.8 mm SL, Python Island, Lake Victoria, Tanzania **B** female *Labrochromismawe* sp. nov. NMBE 1111867, female, 81.6 mm SL, Python Island **C***Labrochromismawepili* sp. nov. holotype, NMBE 1111906, male, 126.2 mm SL, Makobe Island **D***Labrochromismawepili* sp. nov. NMBE 1111912, female, 120.8 mm SL, Makobe Island, Lake Victoria, Tanzania.

The male nuptial coloration revealed no noticeable differences between populations from Python and Kissenda islands. Both populations also shared the same melanin pattern with few very broad vertical bars but total absence of midlateral and dorsolateral bands, whereas interrupted midlateral and dorsolateral bands present in all individuals of the Makobe population (Fig. 2). The absence of midlateral and dorsolateral bands in fish from Python and Kissenda islands and the expression of these bands in fish from Makobe Island was visible in preserved specimens too (Fig. 3). The Kruskal-Wallis tests on the proportional measurements of the populations from Kissenda, Python, and Makobe islands indicated a significant difference among the groups. Further exploration through post hoc tests, locate pronounced differences in multiple traits between the population from Makobe Island and both other populations (Table 2).

**Table 2.** Results of the Kruskal-Wallis comparison test (KW) and Dunn’s posthoc (Bonferroni) for the proportional measurements of Python, Kissenda and Makobe populations. The loadings for Principal Component Analysis (PC1 & PC2) and for Discriminant Function Analysis (DF1 & DF2) of the residuals of linear regressions of each distance against standard length. The adjusted p-value (after Bonferroni correction) for comparison between populations are provided for each trait. Significant p-values after correction are bold and whenever no significant differences are detected by Kruskal-Wallis test, NS is indicated for both population comparisons to denote that post-hoc (Bonferroni) tests were not performed.

Morphometric measurements traits	Python and Kissenda (*L.mawe* sp. nov.)	Python and Makobe (*L.mawe* vs *L.mawepili*)	Kissenda and Makobe (*L.mawe* vs *L.mawepili*)	Kruskal Wallis (KW)	P-value	PC1	PC2	DF1	DF2
SL (mm)	NS	NS	NS	3.8358	0.1469				
BD	0.42824	0.01143	0.01224	6.3051	**0.0427**	0.36640701	-0.21177428	0.39288745	-0.2415679
HL	0.91001	0.01680	0.01988	9.1562	**0.0103**	-0.30908718	0.11330820	-0.4973120	-0.3691148
HW	0.33257	0.01170	0.01279	7.0932	**0.0288**	0.26172810	0.43947012	0.5082432	0.4798611
CPL	NS	NS	NS	5.1290	0.0770	0.28868712	0.18613928	-0.4998712	-0.3863816
CPD	1.00000	0.02484	0.02342	9.0352	**0.0109**	0.27071093	-0.41657362	0.3560223	0.6240751
SnL	0.13211	0.00309	0.00401	9.0552	**0.0108**	-0.22564143	-0.09977733	-0.4749950	-0.6112960
SnW	0.05103	0.02345	0.30001	6.9826	**0.0305**	0.06375188	-0.45124306	0.1672694	-0.7952798
POD	0.04277	0.13225	0.51284	7.1428	**0.0281**	-0.29447601	-0.4028297	0.5851777	1.2259793
EyL	NS	NS	NS	5.1182	0.0774	-0.01427275	-0.47965171	-0.9974223	-0.0553676
EyD	NS	NS	NS	8.8554	0.0621	-0.24497664	-0.21387251	0.7930468	1.2020115
ChD	0.06220	0.03240	0.34310	16.2450	**0.0030**	0.29860841	0.48404488	-0.9532856	0.5843687
LJL	NS	NS	NS	1.6541	0.4373	0.02723807	0.08215604	-0.7268303	0.2668128
LJW	0.91000	0.01450	0.01130	17.4940	**0.0022**	0.35659496	0.35183019	0.8688871	0.8266514
IOW	1.00000	0.01220	0.00701	19.3730	**0.0014**	0.36035519	0.07944776	0.9985393	0.4749023

The Mann-Whitney U tests for intraspecific sexual dimorphism in the morphometric traits showed no significant sexual dimorphism in the measured morphometric traits in any of the populations (Kissenda, Python, and Makobe islands). The p-values for the traits ranged from 0.068 to 0.787, all of which exceeded the threshold for significance (p > 0.05).

**Figure 4.**
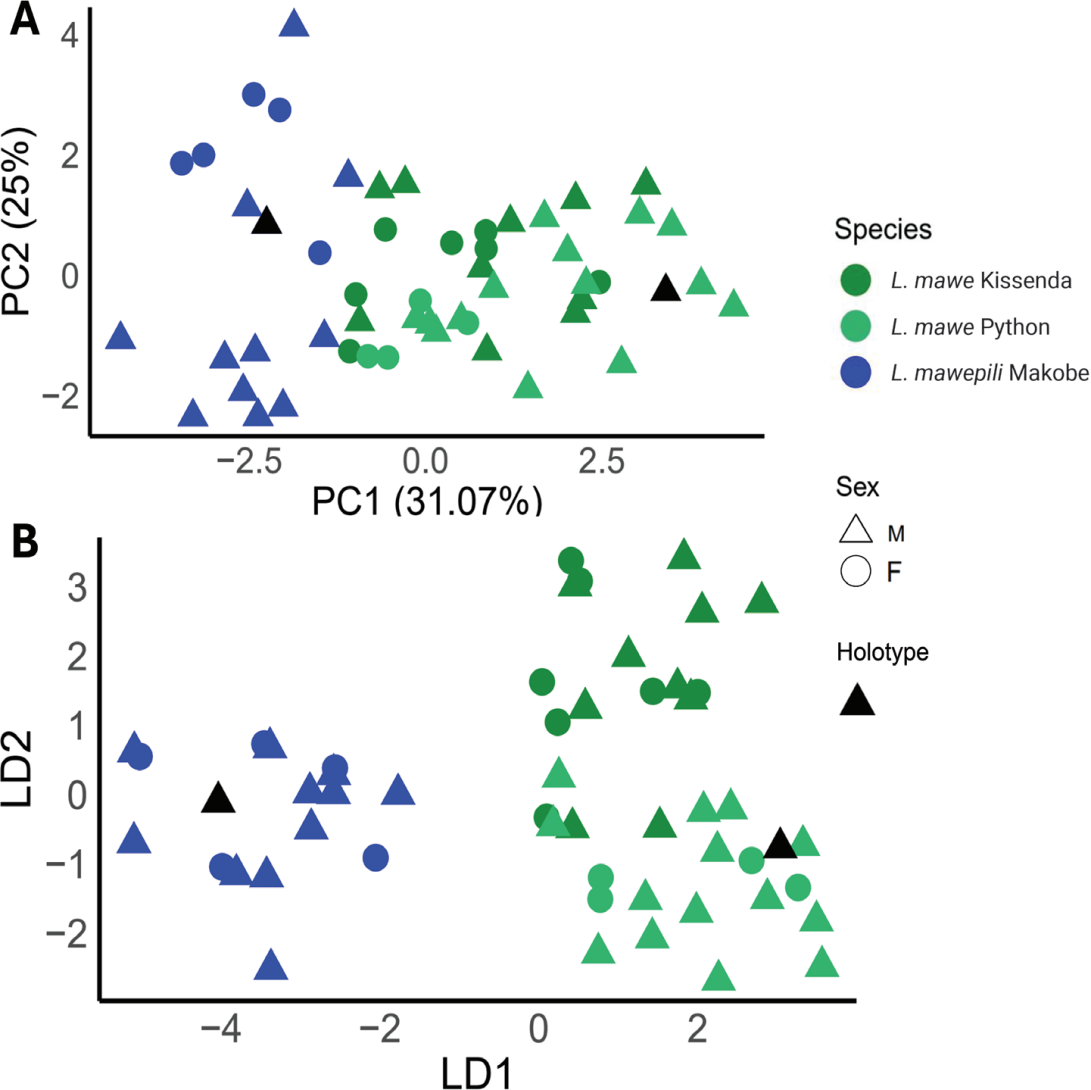
**A**PCA and **B**DFA results on the residuals from linear regressions against standard length of 16 traits.

Principal Component Analysis (PCA) on the residuals of linear regressions of each trait against SL clearly separated the population of Makobe Island from those of Python and Kissenda islands along PC1 (31.07% of variance) whereas none of the other PC axes separated the populations (Fig. 4A). Most variables loaded positively on PC1, with residuals variation in BD, IOW, ChD, and LJW having the largest positive loadings. Variables with significant negative loadings on PC1 included HL and POD. Principal Component 2 (13.12% of variance) was primarily determined by HW and ChD with positive loadings, while EyL, SnW, and CPD contributed negatively (Table 2). However, PC2 did not separate any of the populations. The results of the multivariate analysis of variance (MANOVA) suggest substantial differences along the PC1 between the species, i.e., the populations from Python and Kissenda versus that from Makobe Island (Pillai’s trace statistic = 0.78474, F statistic = 16.143, num Df = 4, den Df = 100, p-value = 3.135e-10).

Discriminant Function Analysis (DFA) on the residuals of linear regressions of each trait against SL corroborates the major differences between the population from Makobe versus both other populations which is all captured by DF1. It additionally reveals small differences between the two latter populations, and this is captured by DF2 (Fig. 4B). The variance explained by DF1 was 78.74%, primarily determined by IOW, LJW and POD. DF2 explained 21.25% of the variance and was determined by ChD and HW.

**Figure 5.**
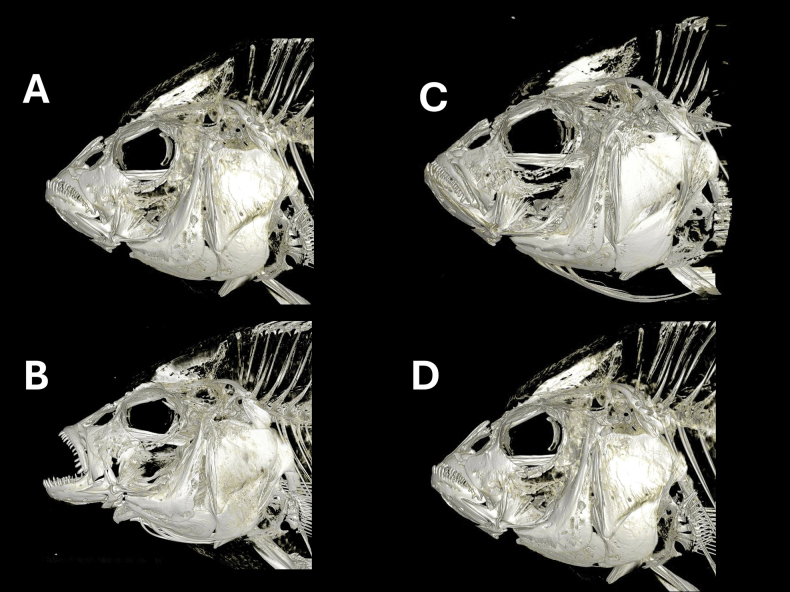
CT scans of the skull of *Labrochromismawe* sp. nov. and *Labrochromismawepili* sp. nov. All are males from top left **A***L.mawe* sp. nov., NMBE 1111873, Python Island **B***L.mawe* sp. nov., NMBE 1111871, Python Island **C***L.mawe* sp. nov., NMBE 1111893, Kissenda Island **D***L.mawepili* sp. nov., NMBE 1111906, Makobe Island, Lake Victoria, Tanzania.

**Figure 6.**
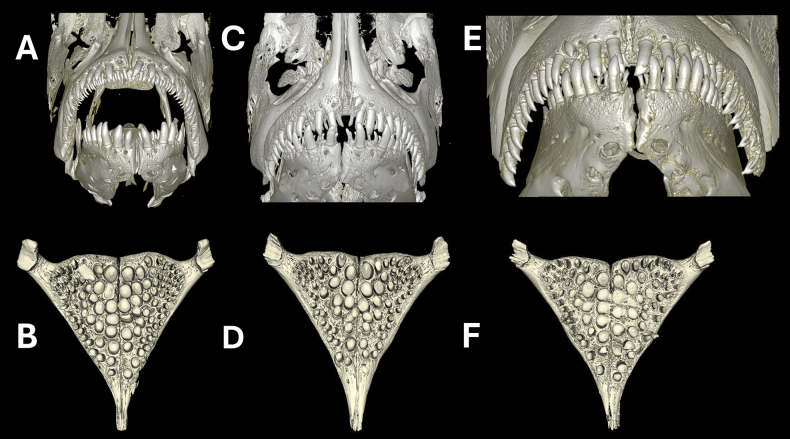
CT scans of the frontal aspect of oral jaws (top) and occlusial aspect of the lower pharyngeal jaws (bottom) of *Labrochromismawe* sp. nov. and *L.mawepili* sp. nov. All are males. From top left, **A, B** (NMBE 1111873) *L.mawe* sp. nov., Python Island **C, D** (NMBE 1111893) *L.mawe* sp. nov., Kissenda Island **E, F** (NMBE 1111906) *L.mawepili* sp. nov., Makobe Island.

CT scans of the skull (Fig. 5) and jaws (Fig. 6) revealed no visible differences in the oral and pharyngeal jaws between individuals from populations of Python and Kissenda islands. Individuals from Makobe Island possess slightly finer and procumbently implanted teeth in the outer row of oral jaws. Based on all the phenotypic data combined, we conclude that the populations from Python and Kissenda islands constitute populations of a single species. We hereby describe this species as *Labrochromismawe* sp. nov.

The population from Makobe is distinct from both the populations from Python and Kissenda Islands in several morphometric traits including shallower BD, narrower IOW and smaller ChD (Table 1), in the presence of mid and dorsolateral stripes but often more muted expression of vertical bars (Figs 2, 3) and they have somewhat finer and somewhat more procumbently implanted teeth in the outer row of the oral jaws. Fish resembling the population from Makobe also occur at Ruti, Igombe and Hippo islands (Seehausen 1996) and we consider these conspecific with those from Makobe. Fish from Ruti, Igombe, and Hippo islands were not included in our current study, as specimens from these locations were not available for investigation. Therefore, we cannot definitively determine if these fish belong to the same species as those from Makobe. This will have to be resolved by future work. We describe this species as *Labrochromismawepili* sp. nov.

### ﻿ Labrochromis mawe sp. nov.

Taxon classificationAnimaliaPerciformesCichlidae

61C41078-60B9-5EC0-B15F-CBBE036CF2F7

https://zoobank.org/E9A00634-AB01-44A0-865D-1A5950FCD0EF

Haplochromis (Labrochromis) “stone”: Witte et al. 1992; Seehausen 1996; Seehausen and Bouton 1996; Seehausen et. al. 1997; Seehausen et al. 1999; Seehausen and van Alphen 1999.
“Haplochromis” “stone”: Bouton et al. 1997.

#### Type material.

***Holotype*.** • NMBE 1111880, male, 127.8 mm SL, Lake Victoria, Python Island, Mwanza Gulf, Tanzania, O. Selz, July 2010. ***Paratypes*.** • 35 Specimens 77.6–124.3 mm SL. All specimens are from Lake Victoria, Tanzania, Mwanza Gulf. • NMBE 1111864, 104.4 mm SL, NMBE 1111865, 91.3 mm SL, NMBE 1111866, 86.2 mm SL and NMBE 1111867, 81.6 mm SL, 4 females, Python Island, O. Seehausen, 01^st^ Dec 1993. • NMBE 1111868 116.0 mm SL, NMBE 1111869, 107.0 mm SL, NMBE 1111881, 112.7 mm SL and NMBE 1111878, 115.6 mm SL, 4 males, Python Island, O. Selz, 21^st^ June 2010. • NMBE 1111870, 90.5 mm SL and NMBE 1111872, 85.5 mm SL, 2 males, Python Island, O. Seehausen, 01^st^ Dec 1993. • NMBE 1111871, 116.7 mm SL and NMBE 1111873, 113.6 mm SL, 2 males, Python Island, O. Seehausen, 12 Nov. 1995. • NMBE 1111886, 120.0 mm SL, NMBE 1111887, 105.0 mm SL and NMBE 1111888, 106.1 mm SL, 3 females, Kissenda Island, O. Selz, 9^th^ June 2010. • NMBE 1111883, 111.5 mm SL and NMBE 1111897, 95.2 mm SL, 2 females, Kissenda Island, O. Selz, 9^th^ June 2010. • NMBE 1111891, 124.3 mm SL and NMBE 1111898, 108.3 mm SL, 2 males, Kissenda Island, O. Selz, 9^th^ June 2010. • NMBE 1111894, 115.0 mm SL, one male, Kissenda Island, O. Selz 7^th^ July 2010. • NMBE 1111895, 91.1 mm SL, one male, Kissenda Island, O. Selz 13^th^ July 2010. • NMBE 1111893, 106.1 mm SL, one male, Kissenda Island, O. Selz, 9^th^ June 2010. • NMBE 1111884, 99.1 mm SL, 1 female, Kissenda Island, O. Selz, 9^th^ June 2010. • NMBE 1111882, 115.7 mm SL, 1 male, Python Island, O. Selz 21^st^ June 2010. • NMBE 1111876, 115.2 mm SL, one male, Python Island, O. Selz, 17^th^ July 2010. • NMBE 1111877, 121.3 mm SL, one male, Python Island, O. Selz, 10^th^ July 2010. • NMBE 1111892, 82.8 mm SL, one male, Kissenda Island, O. Selz, 13^th^ July 2010. • NMBE 1111899, 96.0 mm SL, one male, Kissenda Island, O. Selz, 13^th^ July 2010. • NMBE 1111885, 99.2 mm SL, one female, Kissenda Island, O. Selz, 9^th^ June 2010. • NMBE 1111879, 111.0 mm SL, 1 male, Python, 10^th^ July 2010. • NMBE 1111874, 77.6 mm SL, one male, Python Island, O. Seehausen, 01^st^ Dec 1993. • NMBE 1111875, 87.8 mm SL, one male, Python Island, O. Selz, 10^th^ July 2010. • NMBE 1111890, 103.0 mm SL, 1 male, Kissenda Island, O. Selz, 13^th^ July 2010. • NMBE 1111896, 113.3 mm SL, one male, Kissenda Island, O. Selz, 1^st^ July 2010. • NMBE 1111889, 86.3 mm SL, one male, O. Selz, 14^th^ Nov. 2014.

#### Description.

Based on 36 specimens from Python and Kissenda islands including the holotype. Morphometric, meristic, and dentition characters are given in Table 1.

***Habitus*.** Large growing robust and deep-bodied species with a blunt and wide head, few wide bars and conspicuous red (in males) or brown (females) maculae in the soft part of dorsal fin. Dorsal head profile decurved to moderately concave with heavy head appearance. Snout slightly longer than broad, the mouth oblique, and the lips not thickened. Lateral snout outline with isognathous jaws and obtuse.

***Oral teeth*.** The teeth in the outer tooth row (Fig. 6A, C) are unicuspid to weakly bicuspid, short, stout, distantly spaced, and implanted in an upright position. The inner series teeth are small, tricuspid, and are arranged in two rows anteriorly and anterolaterally in both jaws.

***Dental arcade and tooth band*.** Dental arcade rounded, not square shaped. Inner rows in both jaws are usually separated from the outer row by a moderate gap.

***Lower pharyngeal bone and dentition*.** The lower pharyngeal bone is strongly hypertrophied and stout with strongly enlarged molariform pharyngeal teeth (Fig. 6B, D).

***Scales and squamation*.** The flank is entirely scaled with ctenoid scales. Scales are ovoid with vertical long axis, slender and slightly higher than wide. Operculum: scaled, scales cycloid; cheek: fully scaled, scales cycloid; caudal peduncle: scales moderately ctenoid, chest: entirely scaled; scales cycloid, somewhat deeply embedded, though not as deeply as in many other rock-dwelling cichlids such as *Neochromisgreenwoodi* Seehausen & Bouton, 1998 and *Mbipiambipi* Seehausen, Lippitsch & Bouton, 1998 and smaller than on flank. The size transition to the chest is gradual. The belly; is fully scaled, scales moderately ctenoid and not distinctly smaller than on flanks. Dorsal fin; scaleless, caudal fin; partially scaled with cycloid scales, anal fin; scaleless and pectoral fin scaleless.

***Coloration*.** Melanin pattern in both sexes: Males and females exhibit four to six broad vertical bars on their flanks (note that these can be hard to see in preserved specimens that have bleached slightly). Preserved specimens of both sexes exhibit a brownish coloration, with some individuals retaining their vertical bars (Fig. 3A). Male nuptial coloration: *Labrochromismawe* sp. nov. exhibits male nuptial color polymorphism and found in two distinct color morphs. One morph is entirely blue on the flanks, including the spinous part of the dorsal fin (Fig. 2A, D). The other morph is reddish on the dorsal head surface, and red on operculum, anterior flanks and anterior dorsum with the remaining flanks appearing yellowish and greyish towards the posterior dorsum (Fig. 2B, E; Seehausen and Bouton 1996; Seehausen et. al. 1999). Both morphs possess numerous characteristic red maculae in the soft part of the dorsal fin (Fig. 2). A nape band, nostril, forehead, supraorbital, intraorbital, hind-eye, preopercular and lachrymal stripes (Seehausen et al. 1999) may be visible or not depending on behavioral context; lower lip with a blue sheen. The caudal fin is translucent grey with red streaks and maculae, grading to solid red towards the edge, while the anal fin is proximally blue grey, distally faint red to solid red, with 2–6 orange egg dummies. In the red morph, the red color can extend into the spinous part of the dorsal fin. Females have yellowish-brown coloration.

#### Distribution and ecology.

**Figure 7.**
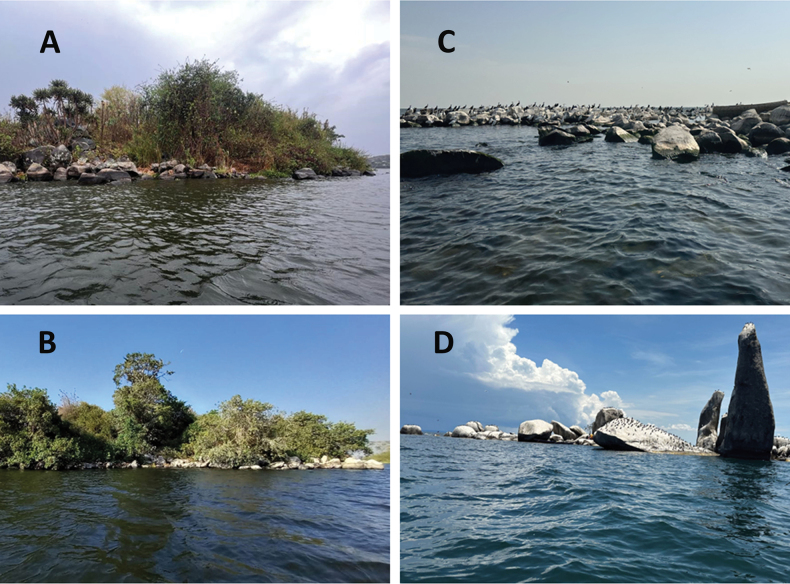
Photographs of the sampling sites of *Labrochromismawe* sp. nov. (**A, B**) and *Labrochromismawepili* sp. nov. (**C, D**) **A** Kissenda Island **B** Python Island **C** Makobe Island and **D** Ruti Island.

*Labrochromismawe* sp. nov. is known from the northern and central Mwanza Gulf in Lake Victoria, Tanzania. The species has been collected at Anchor, Kissenda, Kilimo, Python, Gabalema, Nyamatala and Hippo islands, Bwiru point, Karumo bay, Nyegezi rocks, Amranda point, Ngoma point, and Capri point (Fig. 1). The species inhabits moderately steep to steep slopes, with medium sized to very large rock boulders (Fig. 7). Subadult individuals are commonly caught in shallow inshore waters between rock boulders while adults inhabit greater depths. At the moderately steep Python Islands, they are found somewhat offshore at depths ranging from 3 meters to at least 8 meters. In very steeply sloping areas, such as Anchor Island and Nyegezi rocks, they are located inshore and at depths starting from 1.5 meters downwards (Seehausen 1996).

#### Food.

The diet of *Labrochromismawe* sp. nov. is predominantly snails but insect larvae are eaten as well (Bouton et al. 1997; Seehausen 1996).

#### Breeding.

Like all Lake Victoria haplochromines, *Labrochromismawe* sp. nov. is a female mouthbrooding care giver, wherein the female carries her eggs as well as the hatched larvae inside her mouth, providing them with a safe and nurturing environment until the larvae have resorbed the yolk sac and develop into independent, free-swimming juveniles. Spawning takes place throughout the year as far as we could ascertain. Mouthbrooding females are probably residing in the deeper part of the depth range and are not usually observed among shallow water boulders.

#### Diagnosis and affinities.

The nature of its pharyngeal dentition places *Labrochromismawe* sp. nov. on the same level of structural modification as other described *Labrochromis* species (*L.ishmaeli*, *L.humilior*, *L.pharyngomylus*, *L.teegelaari*, *L.mylergates*, *L.ptistes*, *L.mawepili* sp. nov.). *Labrochromismawe* sp. nov. differs from all other species except *L.mawepili* sp. nov. by eye size (smaller among compared species (21.3–25.9 vs 23–33), (Table 3), and habitat association. Specifically, *L.mawe* sp. nov. restricted to rocky substrates, unlike other species inhabiting sand and/or mud bottoms. *L.mawe* sp. nov. possess shorter head (31.7–34.9% SL) compared to *L.ptistes* (34.2–37.6% SL) and narrower interorbital width (25.0–30.2% HL) compared to *L.mylergates* (26.0–33.0% HL). *L.mawe* sp. nov. and *L.mawepili* sp. nov. share the habitat (rocky substrate) but *L.mawe* sp. nov. possess shorter and stouter teeth than *L.mawepili* sp. nov., wider interorbital width (24.0–31.8% HL) as *L.mawepili* sp. nov. 23.3–27.9 (means 24.6–25.4) % HL, and broader lower jaw (22.7–34.9% HL) as *L.mawepili* sp. nov. (20.2–27.5% HL). *L.mawe* sp. nov. possesses 31 or 32 scales in the lateral line whereas *L.mawepili* sp. nov. 32 or 33 scales (Table 1). *L.mawe* sp. nov. exhibits broad vertical bars and no traces of lateral stripes while *L.mawepili* possesses broken midlateral and dorsolateral bands that are typically as distinct as or more distinct vertical bars. *L.mawe* sp. nov. exhibits color polymorphism with two color morphs (Seehausen and Bouton 1996): one morph is entirely blue on the flanks, including the spinous part of the dorsal fin. The other morph is reddish on the dorsal head surface, and red on operculum, anterior flanks and anterior dorsum with the remaining flanks yellow and greyish towards the posterior dorsum. Both morphs possess numerous characteristic red maculae on the soft part of the dorsal fin. The caudal fin is translucent grey with red streaks and maculae, grading to solid red towards the edges. The anal fin is proximally blue grey, distally faint red to solid red, with 2–6 orange egg dummies. Male nuptial coloration of *L.mawepili* sp. nov. is comparable to the blue morphy of *L.mawe* sp. nov. However, *L.mawepili* sp. nov. possesses 5–10 orange egg dummies in the anal fin. Male nuptial colouration of *L.pharyngomylus* is blue-grey overlying silver, with a distinct coppery sheen on the flanks. The dorsal fin is hyaline with pinkish lappets and a pink margin in the soft part. The anal fin is hyaline with yellow egg dummies, while the caudal fin is hyaline with a pink flush, most intense distally and on the ventral half of the fin (Greenwood 1981). The male nuptial coloration of *L.humilior* is dark silvery-grey with intense dusky blotches. A coppery flush extends over the cheek, operculum, and flank up to the origin of the anal fin. The anal and caudal fins are light red, with the color intensifying along the margins of both fins. The anal fin bears two or three yellow egg dummies (Greenwood 1981). Breeding males of *L.teegelaari* have a purplish-grey dorsum, with the purple color more intense anteriorly. The flanks, chest, and belly are bright red, while the caudal peduncle is yellow with a faint red overlay. Traces of 4–6 vertical bars are visible on the flanks. The dorsal head surface is grey with a red flush, and the rest of the head is bright red, except for the lower lip and branchiostegal membrane, which are white. The lachrymal bar is faint, and a dark bar is present on the vertical preopercular limb. The dorsal fin is light grey with a faint red flush, dark grey lappets, and red maculae on the soft part. The anal fin is light red anteriorly and greyish posteriorly, with egg dummies orange to reddish. A nape band, supraorbital, intraorbital, hind-eye, preopercular and lachrymal stripes faint; the caudal fin is hyaline, yellowish proximally, and has red maculae and streaks (Greenwood 1981).

**Table 3.** Morphometric character comparison of *L.mawe* sp. nov. and *L.mawepili* sp. nov. with other described *Labrochromis* species and ‘*H.theliodon*’ from Lake Victoria (Greenwood 1981).

Species		*L.teegelaari*	*L.mylergates*	*L.humilior*	*L.pharyngomylus*	*L.ptistes*	*L.ishmaeli*	‘*H.theliodon*’	*L.mawe* sp. nov.	*L.mawepili* sp. nov.
SL (mm)	Range	74.0–100.5	102.0–137.0	65.0–90.0	70.0–126.0	90.0–106.0	82–136.0	75.0.0–95.0	77.6–127.8	80.7–144.2
BD%SL	Range	39.0–43.0	38.0–45.0	29.0–37.5	33.8–42.0	38.6–42.0	37.0–45.5	35.7–38.6	36.1–44.0	34.5–42.7
	Mean	41.0	42.0	34.4	38.5	40.0	40.1	37.4	40.2	38.0
HL%SL	Range	32.0–36.0	33.0–37.0	31.6–37.8	31.5–36.8	34.2–37.6	33.8–37.5	33.7–37.3	31.7–35.8	32.6–37.0
	Mean	34.0	34.9	34.7	34.6	36.0	34.8	36.4	33.3	34.6
POD%HL	Range	14.0–19.0	12.0–20.0	13.6–17.9	13.8–19.0	14.7–17–4	15.3–20.5	15.3–20.5	15.4–21.2	16.8–19.7
	Mean	17.0	16.0	16.3	16.8	16.4	17.0	17.3	17.2	18.0
IOW%HL	Range	25.0–30.0	26.0–33.0	21.0–28.6	23.7–28.5	23.5–26.0	24.0–32.0	22.5–25.8	25.0–31.8	23.3–27.9
	Mean	27.0	29.0	24.2	26.3	24.7	27.6	24.6	27.9	25.1
SnL%HL	Range	27.0–31.0	28.0–33	27.0–34.8	27.3–33.3	29.4–32.4	29.0–36.0	32.2–35.5	27.8–36.7	30.7–36.0
	Mean	29.0	31.0	30.9	30.8	30.6	31.6	33.7	31.1	32.2
ChD%HL	Range	19.0–25.0	20.0–29.0	18.5–23.2	19.7–27.0	20.5–24.3	20.7–31.0	21.4–27.0	21.7–29.3	22.2–30.1
	Mean	22.0	23.0	21.2	24.1	22.0	25.5	24.3	25.8	26.2
LJL%HL	Range	33.0–40.0	35.0–43.0	33.4–39.6	35.8–44.0	37.3–41.2	35.8–42.5	37.4–39.3	35.2–44.2	32.9–42.0
	Mean	37.0	39.0	36.6	38.6	39.0	39.1	38.2	39.5	38.8
EyL%HL	Range	27.0–33.0	28.0–33.0	27.0–32.5	23.0–31.8	26.5–32.4	23.0–31.0	24.1–26.8	21.3–25.9	18.7–25.8
	Mean	30.0	31 .0	30.3	26.5	30.0	27.7	25.2	22.9	22.9

#### Etymology.

Species name *mawe* from the Swahili word, for stone, referring to the habitat occupation of the species that is confined to rocks.

### ﻿ Labrochromis mawepili sp. nov.

Taxon classificationAnimaliaPerciformesCichlidae

947426EF-975F-5C08-9D86-29D89116E173

https://zoobank.org/1EA9E503-6957-40E9-9D89-3F03374EFEE5

Haplochromis (Psammochromis) "striped crusher": Seehausen 1996; Seehausen et al. 1997.
Haplochromis "striped crusher": Seehausen and Bouton 1998.
Labrochromis sp. "stone": Karvonen et al. 2018; Gobbin et al. 2020, 2021; Meier et al. 2023.

#### Type material.

***Holotype*.** • NMBE 1111906 mature male, 126.2 mm SL, Lake Victoria, Makobe Island, Speke Gulf, Tanzania, O. Selz, July 2010. ***Paratypes*.** • 16 specimens, 80.8–144.2 mm SL. All specimens are from Lake Victoria, Makobe Island, Speke Gulf, Tanzania. • NMBE 1111916, 105.4 mm SL, one male, O. Seehausen & S. Mwaiko, 2005. • NMBE 1111917, 80.8 mm SL, one female, O. Selz, 6^th^ August, 2010. • NMBE 1111908, 110.6 mm SL, one female, O. Selz, 6^th^ August, 2010. • NMBE 1111914, 120.1 mm SL, one female, O. Selz, 21^st^ July, 2010. • NMBE 1111911, 123.3 mm SL, one male, O. Selz, 3^rd^ June, 2010. • NMBE 1111909, 120.8 mm SL, one female, O. Selz, 23^rd^ June, 2010. • NMBE 1111907, 99.8 mm SL, one male, O. Selz, 23^rd^ July, 2010. • NMBE 1111910, 101.3 mm SL, one male, O. Selz, 26^th^ July, 2010. • NMBE 1111905, 98.1 mm SL, one male, O. Selz, 3^rd^ June, 2010. • NMBE 1111912, 120.8 mm SL, one female, O. Selz, 23^rd^ July, 2010. • NMBE 1111913, 88.2 mm SL, one male, O. Selz, 23^rd^ July, 2010. • NMBE 1111900 116.7 mm SL, one male, O. Selz, 8^th^ August, 2010. • NMBE 1111901 144.2 mm SL, O. Selz, 5^th^ August, 2010. NMBE 1111902, 134.9 mm SL, one male, O. Selz, 2^nd^ August, 2010. • NMBE 1111904, 112.6 mm SL, one male, O. Selz, 23^rd^ July, 2010. • NMBE 1111903, 106.8 mm SL, one male, O. Selz, 23^rd^ July, 2010.

#### Description.

Based on 17 specimens from Makobe Island, Lake Victoria, Tanzania (Table 1) including the holotype.

***Habitus*.** Large growing robust species, relatively deep-bodied with broken mid and dorsolateral bands and vertical bars creating a broken chessboard pattern in both sexes. The dorsal head profile is straight to moderately concave with a heavy head appearance. Oblique mouth with neither enlarged nor thickened lips.

***Oral teeth*.** Teeth in the outer tooth row slender, unicuspid to weakly bicuspid, distantly spaced, slightly recurved, their implantation in the lower jaw is somewhat procumbent (Fig. 6E). The inner series teeth are small, tricuspid, and are arranged in two rows anteriorly and anterolaterally in both jaws

***Dental arcade and tooth band*.** Dental arcade rounded, not square shaped. Inner rows in both jaws usually separated from the outer row by a moderate gap. Two rows of inner teeth, anteriorly and anterolaterally in both jaws.

***The lower pharyngeal bone jaw and dentition*.** The lower pharyngeal jaw is strongly hypertrophied and stout with strongly enlarged molariform pharyngeal teeth (Fig. 6F).

***Scales and squamation*.** The flank scales are ctenoid, ovoid with vertical long axis, slender and somewhat higher than wide. Operculum; scaled with cycloid scales, cheek; fully scaled with cycloid scales, caudal peduncle; fully scaled, scales moderately ctenoid, the chest; is fully scaled with smaller cycloid scales compared to those on the flanks somewhat deeply embedded although not as deeply as in many other rock-dwelling cichlids. The size transition is gradual. The belly is entirely scaled, with scales moderately ctenoid and not distinctly smaller than on flanks and somewhat deeply embedded. Dorsal fin; scaleless, caudal fin; partially scaled with cycloid scales, anal fin scaleless and pectoral fin; scaleless.

***Coloration*.** Melanin pattern in both sexes: Both adults and subadults exhibit a broken dorso lateral and broken mid-lateral bands, together with the 4 vertical bars creating a broken chessboard pattern. The broken chessboard pattern is more prominent in females and subadult males (Fig. 2H, I) while adult males more often have the lateral bands purely expressed (Fig. 2G). Male nuptial coloration: Adult males are metallic blueish on the posterior flanks, the mid flanks are purplish, anterior and posterior dorsum are greyish (Fig. 2). Dorsal fin is blue grey with numerous red maculae within the soft part of the fin. A nape band, supraorbital, intraorbital, hind-eye, preopercular and lachrymal stripes faint; lower lip with a greenish sheen. The caudal fin is blue grey with red streaks and maculae, and the anal fin is proximally blue grey, distally faint red with 5–10 orange egg dummies (Fig. 2). Preserved specimens of both sexes are brownish and retain their broken longitudinal bands and fin maculae.

#### Distribution and ecology.

*Labrochromismawepili* sp. nov. is exclusively known from the Speke Gulf in Lake Victoria, Tanzania with one observation from the northern Mwanza Gulf. The species has been observed at Makobe, Ruti, Igombe, and Hippo islands (Fig. 1) The species predominantly inhabits offshore areas beyond 4 meters in water depth with gentle to modest slopes. At steeply sloping islands it is often associated with the gently sloping small boulders habitat at the base of the larger and steeply sloping rocks (Fig. 7) in depths of 10 meters and beyond. Females of *Labrochromismawepili* sp. nov. are more commonly encountered than males, distinguishing it as one of the few rock-dwelling haplochromines with such a prevalence. Despite (or perhaps because of) sharing similar habitat affinities as *L.mawe* sp. nov., sympatry between these two species is rare and is only known from Hippo Island where *L.mawe* sp. nov. was frequently encountered in crevices among inshore rocks, while *L.mawepili* sp. nov. was only encountered once, ~ 10 m offshore in deeper water (Seehausen 1996). *Labrochromismawepili* sp. nov. coexists with *Astatoreochromisalluaudi* at all islands, but while *L.mawepili* sp. nov. occupies offshore areas beyond 4 meters depth, *A.alluaudi* tends to be more common in shallower waters.

#### Food.

*Labrochromismawepili* sp. nov. is primarily preying on snails and ostracods, with occasional consumption of insect larvae, albeit to a lesser extent (Seehausen 1996).

#### Breeding.

As for *Labrochromismawe* sp. nov.

#### Diagnosis and affinities.

*Labrochromismawepili* sp. nov. shares a comparable structural modification in pharyngeal dentition with other described *Labrochromis* species (*L.ishmaeli*, *L.humilior*, *L.pharyngomylus*, *L.teegelaari*, *L.mylergates*, *L.ptistes*, *L.mawe* sp. nov.) and shares its rocky habitat association with *L.mawe* sp. nov. It differs from *L.ishmaeli*, *L.humilior*, *L.pharyngomylus*, *L.teegelaari*, *L.mylergates*, *L.ptistes*, *L.mawe* sp. nov. in color patterns and habitat association. *Labrochromismawepili* sp. nov. exhibit broken longitudinal stripes and vertical bars while *L.mawe* sp. nov. displays vertical bars without longitudinal stripes. Male nuptial coloration of *L.mawepili* sp. nov. are shared with those of the blue morph of *L.mawe* sp. nov. (See above in diagnosis of *L.mawe* sp. nov.). *L.mawepili* sp. nov. possess a narrower interorbital width (23.3–27.9% HL) than *L.mawe* sp. nov. (24.0–31.8% HL), *L.teegelaari* (25.0–30.0% HL) and *L.mylergates* (26.0–33.0%HL). Longer snout (30.7–36.0% HL) than *L.ptistes* (29.4–32.4% HL), *L.pharyngomylus* (27.3–33.3% HL), *L.humilior* (27.0–34.8% HL), *L.mylergates* (28.0–33% HL) and *L.teegelaari* (27.0–31.0); (Table 3; Greenwood 1980). *Labrochromismawepili* sp. nov. possess smaller eyes (18.7–25.8% HL) than ‘*H.’ theliodon* (24.1–26.8% HL), *L.ishmaeli* (23.0–31.0), *L.ptistes* (26.5–32.4% HL), *L.pharyngomylus* (23.0–31.8% HL), *L.humilior* (27.0–32.5% HL), *L.mylergates* (28.0–33.0% HL), *L.teegelaari* (27.0–33.0% HL), and slightly smaller than *L.mawe* sp. nov. (21.3–25.9).

#### Etymology.

Species name *mawepili*, from Swahili, *mawe* means stone and *pili* means second. Refers to similarity in habitat association between this species and *L. mawe* and the superficial resemblance with the latter.

## ﻿Discussion

*Labrochromismawe* sp. nov. and *L.mawepili* sp. nov. are unique among described *Labrochromis* species in their tight association with rocky habitats, a feature not observed in other described *Labrochromis* species (Greenwood 1981; Seehausen 1996). Additionally, these newly described species exhibit smaller eyes than all previously described species, probably because they live in better illuminated habitat. A recent study using phylogenomic analysis found that all five *Labrochromis* species from Lake Victoria for which whole genomes were available to the authors formed a single, monophyletic and statistically supported clade (Meier et al. 2023). *L.mawepili* sp. nov. (but labeled *L.* sp. "stone") was inluded in that Study. While previous studies have exclusively described *Labrochromis* species inhabiting sandy and soft substrates, the description of these two species highlights the ecological diversity within the lineage and the role of habitat specialization in this adaptive radiation. Fairly extensive lake-wide sampling of our team suggests that both new species are either very rare or absent from suitable habitats outside Mwanza and Speke Gulf and may be endemic to these regions. Other *Labrochromis* species occur in rocky habitats elsewhere in the lake and these await taxonomic description.

## Supplementary Material

XML Treatment for Labrochromis mawe

XML Treatment for Labrochromis mawepili

## ﻿Additional information

### Author ORCIDs

Anna Mahulu https://orcid.org/0000-0003-3808-605X

Ole Seehausen https://orcid.org/0000-0001-6598-1434

### Data availability

All of the data that support the findings of this study are available in the main text.

## References

[B1] Barel CND Van Oijen MJP Witte F Witte-Maas ELM (1977) An introduction to the taxonomy and morphology of the Haplochromine Cichlidae from Lake Victoria. Netherlands. Journal of Zoology 27(4): 381–389. 10.1163/002829677X00207

[B2] Bouton N Seehausen O Van Alphen OJJ (1997) Resource partitioning among rock‐dwelling Haplochromines (Pisces: Cichlidae) from Lake Victoria. Ecology Freshwater Fish 6(4): 225–240. 10.1111/j.1600-0633.1997.tb00165.x

[B3] Fedorov A Beichel R Kalpathy-Cramer J Finet J Fillion-Robin JC Pujol S Bauer C Jennings D Fennessy F Sonka M Buatti J Aylward S Miller JV Pieper S Kikinis R (2012) 3D slicer as an image computing platform for the quantitative imaging network. Magnetic Resonance Imaging 30(9): 1323–1341. 10.1016/j.mri.2012.05.001 22770690 PMC3466397

[B4] Fryer G Iles TD (1972) The Cichlid Fishes of the Great Lakes of Africa: Their Biology and Evolution. Oliver and Boyd, Edinburgh.

[B5] Genner MJ Seehausen O Cleary DFR Knight ME Michel E Turner GF (2004) How does the taxonomic status of allopatric populations influence species richness within African cichlid fish assemblages? Journal of Biogeography 31(1): 93–102. 10.1046/j.0305-0270.2003.00986.x

[B6] Gobbin TP Vanhove MPM Pariselle A Groothuis TGG Maan ME Seehausen O (2020) Temporally consistent species differences in parasite infection but no evidence for rapid parasite‐mediated speciation in Lake Victoria cichlid fish. Journal of Evolutionary Biology 33(5): 556–575. 10.1111/jeb.13615 32163649 PMC7318199

[B7] Gobbin TP Vanhove MPM Seehausen O Maan ME Pariselle A (2021) Four new species of *Cichlidogyrus* (Platyhelminthes, Monogenea, Dactylogyridae) from Lake Victoria Haplochromine cichlid fishes, with the redescription of *C.bifurcatus* and *C.longipenis*. Preprint. Zoology: Analysis of Complex Systems, ZACS. 10.1101/2021.01.29.428376 PMC11305117 39109983

[B8] Goldschmidt T Witte F (1992) Explosive speciation and adaptive radiation of Haplochromine cichlids from Lake Victoria: An Illustration of the Scientific Value of a Lost Species Flock. SIL Communications, 1953–1996, 23(1): 101–107. 10.1080/05384680.1992.11904013

[B9] Greenwood PH (1974) The cichlid fishes of Lake Victoria, East Africa: The biology and evolution of a species flock. Bulletin of the British Museum (Natural History). Zoology: Analysis of Complex Systems, ZACS (Supplement 6): 1–134. 10.5962/p.119071

[B10] Greenwood PH (1980) Towards a phyletic classification of the ‘genus’ *Haplochromis* (Pisces, Cichlidae) and related taxa. Part 2; the Species from Lakes Victoria, Nabugabo, Edward, George, and Kivu. Bulletin of the British Museum, Natural History. Zoology 39: 1–101. 10.5962/bhl.part.13268 [Natural History]

[B11] Greenwood PH (1981) The Haplochromine fishes of the East African Lakes: Collected Papers on Their Taxonomy, Biology and Evolution (with an Introduction and Species Index). Kraus International Publications, München. https://www.zvab.com/products/isbn/9783601004837

[B12] Greenwood PH (1984) African Cichlids and Evolutionary Theories. In: Echelle AA, Kornfield I (Eds) Evolution of Fish Species Flocks, 141–154. University Maine, Orono, USA. https://cichlidae.com/reference.php?id=5149

[B13] Hoogerhoud RJC (1984) A taxonomic reconsideration of the Haplochromine genera *Gaurochromis* Greenwood, 1980 and *Labrochromis* Regan, 1920 (Pisces, Cichlidae) 34(4): 539–565. 10.1163/002829684X00281

[B14] Karvonen A Wagner CE Selz OM Seehausen O (2018) Divergent parasite infections in sympatric cichlid species in Lake Victoria. Journal of Evolutionary Biology 31(9): 1313–1329. 10.1111/jeb.13304 29944770

[B15] Katongo C Seehausen O Snoeks J (2017) A new species of *Pseudocrenilabrus* (Perci­formes: Cichlidae) from Lake Mweru in the Upper Congo River System. Zootaxa 4237(1): 181–190. 10.11646/zootaxa.4237.1.10 28264309

[B16] Keller I Wagner CE Greuter L Mwaiko S Selz OM Sivasundar Wittwer AS Seehausen O (2013) Population genomic signatures of divergent adaptation, gene flow and hybrid speciation in the rapid radiation of Lake Victoria cichlid fishes. Molecular Ecology 22(11): 2848–2863. 10.1111/mec.12083 23121191

[B17] Martinez CM Corn KA Williamson S Satterfield D Roberts-Hugghis AS Barley A Borstein SR McGee MD Wainwright PC (2024) Replicated functional evolution in cichlid adaptive radiations. American Naturalist 3(3): 242–257. 10.1086/731477 39179237

[B18] McGee MD Borstein SR Meier JI Marques DA Mwaiko S Taabu A Kishe MA Meara BO Bruggmann R Excoffier L Seehausen O (2020) The ecological and genomic basis of explosive adaptive radiation. Nature 586(7827): 75–79. 10.1038/s41586-020-2652-7 32848251

[B19] Meier JI Marques DA Mwaiko S Wagner CE Excoffier L Seehausen O (2017) Ancient hybridization fuels rapid cichlid fish adaptive radiations. Nature Communications 8(1): 14363. 10.1038/ncomms14363 PMC5309898 28186104

[B20] Meier JI McGee MD Marques DA Mwaiko S Kishe M Wandera S Neumann D Mrosso H Chapman LJ Chapman C Kaufman L Taabu-Munyaho A Wagner C Bruggmann R Excoffier L Seehausen O (2023) Cycles of fusion and fission enabled rapid parallel adaptive radiations in African Cichlids. Science 381(6665): eade2833. 10.1126/science.ade2833 37769075

[B21] Santos ME Lopes JF Kratochwil CF (2023) East african cichlid fishes. EvoDevo 14(1): 1. 10.1186/s13227-022-00205-5 36604760 PMC9814215

[B22] Seehausen O (1996) Lake Victoria Rock Cichlids: Taxonomy, Ecology, and Distribution. Verduyn Cichlids, Zevenhuizen, 304 pp.

[B23] Seehausen O (1999) A Reconsideration of Ecological Composition of the Cichlid Species Flock in Lake Victoria before and after the Nile Perch Boom. In: van Densen WLT, Morris MJ (Eds) Fish and Fisheries of Lakes and Reservoirs in Southeast Asia and Africa, 281–293. Westbury, Ottey, UK. https://boris.unibe.ch/71531

[B24] Seehausen O (2006) African cichlid fish: A model system in adaptive radiation research. Proceedings of the Royal Society B, Biological Sciences 273(1597): 1987–1998. 10.1098/rspb.2006.3539 PMC1635482 16846905

[B25] Seehausen O Bouton N (1996) Polychromatism in rock dwelling Lake Victoria cichlids: Types, distribution, and observation on their genetics. The Cichlids. Yearbook 6: 36–45.

[B26] Seehausen O Bouton N (1998) The community of rock-dwelling cichlids in Lake Victoria. Bonner zoologische Beiträge 47(3–4): 301–311. https://www.zobodat.at/pdf/Bonner-Zoologische-Beitraege_47_0301-0311.pdf

[B27] Seehausen O Witte F Katunzi EF Smits J Bouton N (1997) Patterns of the remnant cichlid fauna in Southern Lake Victoria. Conservation Biology 11(4): 890–904. 10.1046/j.1523-1739.1997.95346.x

[B28] Seehausen O Lippitsch E Bouton N Zwennes H (1998) Mbipi, the rock-dwelling cichlids of Lake Victoria: Description of three new genera and fifteen new species (Teleostei). Ichthyological Exploration of Freshwaters 9(2): 129–228. https://boris.unibe.ch/71539

[B29] Seehausen O van Alphen JJM Lande R (1999) Color polymorphism and sex ratio in a cichlid fish as an incipient stage in sympatric speciation by sexual selection. Ecology Letters 2(6): 367–378. 10.1046/j.1461-0248.1999.00098.x

[B30] Van Rijssel JC Moser FN Mwaiko S Seehausen O (2022) Strong species structure but weak geographical structure in Demersal Lake Victoria cichlids. Ecology and Evolution 12(12): e9669. 10.1002/ece3.9669 PMC9790821 36582774

[B31] Vranken N Van Steenberge M Snoeks J (2020) Similar ecology, different morphology: Three new species of oral‐mollusc shellers from Lake Edward. Journal of Fish Biology 96(5): 1202–1217. 10.1111/jfb.14107 31338837

[B32] Vranken N Van Steenberge M Mbalassa M Snoeks J (2023) Just below the surface, the pelagic haplochromine cichlids from the Lake Edward System. Hydrobiologia 850(14): 3173–3195. 10.1007/s10750-023-05246-y

[B33] Wagner CE Keller I Wittwer S Selz OM Mwaiko S Greuter L Sivasundar A Seehausen O (2013) Genome‐wide RAD sequence data provide unprecedented resolution of species boundaries and relationships in the Lake Victoria cichlid adaptive radiation. Molecular Ecology 22(3): 787–798. 10.1111/mec.12023 23057853

[B34] Witte F van Oijen MJR (1990) Taxonomy, ecology and fishery of Lake Victoria haplochromine trophic groups. Zoologische Verhandelingen, Leide (262): 1–16.

[B35] Witte F Goldschmidt T Wanink JH Van Oijen MJP Goudswaard PC Witte-Maas E Bouton N (1992) The destruction of an endemic species flock: Quantitative data on the decline of the haplochromine cichlids of Lake Victoria. Environmental Biology of Fishes 34(1): 1–28. 10.1007/BF00004782

[B36] Witte F Seehausen O Wanink JH Kishe-Machumu MA Rensing M Goldschmidt T (2013) Cichlid species diversity in naturally and anthropogenically turbid habitats of Lake Victoria, East Africa. Aquatic Sciences 75(2): 169–183. 10.1007/s00027-012-0265-4

[B37] Young KA Snoeks J Seehausen O (2009) Morphological diversity and the roles of contingency, chance, and determinism in African cichlid radiations. PLOS ONE 4(3): e4740. 10.1371/journal.pone.0004740 PMC2648897 19270732

